# Education, poverty and "purity" in the context of adolescent girls' secondary school retention and dropout: A qualitative study from Karnataka, southern India

**DOI:** 10.1371/journal.pone.0202470

**Published:** 2018-09-05

**Authors:** Satyanarayana Ramanaik, Martine Collumbien, Ravi Prakash, Lottie Howard-Merrill, Raghavendra Thalinja, Prakash Javalkar, Srikanta Murthy, Ben Cislaghi, Tara Beattie, Shajy Isac, Stephen Moses, Lori Heise, Parinita Bhattacharjee

**Affiliations:** 1 Karnataka Health Promotion Trust (KHPT), IT Park, Rajajinagar, Bangalore, India; 2 Department of Global Health and Development, London School of Hygiene and Tropical Medicine (LSHTM), London, London, United Kingdom; 3 Center for Global Public Health, University of Manitoba, Winnipeg, Manitoba, Canada; 4 Department of Population, Family and Reproductive Health, Johns Hopkins Bloomberg School of Public Health, Baltimore, United States of America; Public Library of Science, UNITED KINGDOM

## Abstract

**Background:**

Gender-related norms and poverty remain important structural barriers to secondary school attendance among adolescent girls in southern India. We analyse how gender norms interact with family deprivation and dynamics to result in girls dropping out of school; we identify the main facilitators of school retention and changes to gender socialisation.

**Methods:**

Longitudinal qualitative case studies with 36 girls were nested within a cluster randomized trial to evaluate the *Samata* intervention targeting adolescent girls in Bagalkote and Vijayapura districts in northern Karnataka. We used two rounds of in-depth interviews, conducted in 2014 at a time when respondents were in 8^th^ standard at the age of 13 to 14 and sixteen months later. We combined thematic and narrative analyses.

**Results:**

Our study found that poverty and socioeconomic realities at the household level strongly affect conformity with discriminatory gender practices such as restricting girls’ mobility. The value placed on education by parents clearly differentiates the regular school goers from those frequently absent and others who dropped out. With active encouragement of the girls’ educational and career aspirations, parents engendered the girl’s agency to communicate openly both at home and at school, allowing subtle changes to gender performance while resisting the pressure of social sanctions. In contrast, where educational aspirations were weak, parents invested more intensely in enforcing correct performance of gender, prioritising her well-being by aiming to secure her future in a good marriage. Among poorer families, girls’ domestic duties came at the cost of schooling with concerns about protecting her sexual purity predominating.

**Conclusions:**

In contexts where a strong gender ideology of virginity before marriage rules, subtle shifts in harmful gender practices are possible. Interventions aiming to improve education need to target the most deprived families, focussing on trust building through open communication.

## Introduction

Inclusive and quality primary and secondary education for boys and girls are critical to sustainable development and gender equality [1]. While there has been substantial progress in expanding universal access to primary education, gender disparities persist and widen in secondary school. Gender socialisation during early adolescence (age 10 to 14) comes with increased expectations for girls and boys to adhere to socially constructed norms and practices [2]. Boys are typically afforded greater independence while more restrictions are placed on girls [3], contributing to early marriage of girls and girls dropping out of secondary school at higher rates than boys. Educational attainment strongly correlates with age of marriage in diverse settings, yet there is little evidence for a direct cause-effect relationship [4]. Among adolescent girls and young women, early marriage and school dropout have many of the same drivers and consequences. Child marriage (marrying before age 18) leads to early child-bearing, which increases adverse reproductive and sexual health outcomes, resulting in higher levels of maternal and child morbidity and mortality, and increased HIV infection [5–7]. Truncated schooling is associated with a range of additional negative outcomes: lower earning potential and access to financial resources, lower use to health services including family planning, higher fertility and lower agency and decision power within households [8–10]. The adverse consequences of poor education persist across a woman’s life-course and into the next generation, perpetuating poverty and ill-health among the poorest [11]. Gender equity in access to and completion of secondary school is thus crucial to halting cycles of adverse life outcomes for girls and their families [12].

A review and analysis of academic and development agency literature on why girls leave school identified a complex array of supply- and demand-side factors [13]. Most studies were cross-sectional and quantitative, leaving many questions about pathways to dropout unanswered. Prospective qualitative designs studying ‘*processes’* of dropout are better suited to understand how individual, family and social structural factors interact to push children out of school. For interventions aiming to increase secondary education, it is equally important to explore what factors facilitate keeping girls in schools in the same disadvantaged contexts [13,14]. In this paper, we study dropout and retention in school among lower caste rural girls age 13–15 in northern Karnataka, with particular attention to how gender norms interact with other social and economic realities in this setting.

### The context of girls’ education in Karnataka

The Government of India programme *Rashtriya Madhyamik Shiksha Abhiyan* introduced various “schemes” to improve access, equity and quality of secondary education [15]. The National Family Health Survey in Karnataka shows progress in the proportion of women who report having completed 10th standard, increasing from 28% of women aged 15–49 in 2005 to 45% in 2015. This reflects a period of rapid economic and social change, with an increased supply of, and demand for, education. However, progress is much slower for socially disadvantaged groups such as girls from scheduled castes and tribes (SC/ST) in rural Northern Karnataka [16,17]. Bagalkote and Vijayapura (earlier known as Bijapura) district have the worst health index ranking in the state [18], poor literacy levels and the highest rate of dropout among SC/ST girls in secondary school [19]. This region is also known for the *Devadasi* tradition, historically involving the dedication of young girls from the scheduled caste to a Goddess through a marriage ceremony, after which she served the temple and, in some cases, provided sexual services for priests and upper caste men [20,21]. Nowadays *Devadasis* engage in commercial sex, devoid of religious duties and social respect. After the Government banned the practice in the 1980s, girls still get dedicated with the first-sex ceremony a lucrative event, followed by work in brothels in major cities in Maharashtra arranged through a *Devadasi* network[22]. Girls whose mothers are *Devadasi* are thus regarded as particularly vulnerable to school dropout.

India is a patriarchal society where gender norms disadvantage women regarding power and resources, subordinating them to men in public and private matters. Gender-related norms also intersect with other significant structural factors, such as poverty, caste, rural residence and customs to limit women’s access to formal education and economic opportunities [23,24]. Compared to their male peer’s girls are disproportionately expected to do domestic chores and take on caring responsibilities at home, limit movement outside the home and remain virgin until marriage. This system of norms has implications for girls’ school dropout and the practice of child marriage.

Parents have primary decision-making power about when and to whom their daughters are married, and base decisions regarding marriage and girl’s participation in education on both economic and social considerations. In rural India, child marriage is a custom consisting of several rituals: after the bride and bridegroom perform the marriage rituals, the girl often remains living in her natal home. The marriage is consummated only after the ceremony of *gauna*, usually years later, when the bride moves to the husband’s house. Uncle–niece marriages are also customary in southern India. In these situations, a man marries his elder sister’s daughter, further strengthening the close kin group [25]. Despite laws in India prohibiting marriage before the age of 18, in practice families tend not to consider an age younger than 18 as a reason to prevent their daughter from being married.

### Theoretical perspectives

Various theoretical perspectives are needed to consider what influences girls’ participation in secondary education as structural factors operate through indirect and complex ways to influence the individual. Beyond empowering girls and building individual assets, an enabling environment needs building at family, community and societal levels [8]. We draw on the capability approach, social norms theory and gender theory to help achieve a more nuanced understanding of pathways to school dropout.

### Capability approach

Amartya Sen’s, Capability Approach calls for a focus on how social context constrains individual freedoms and is central to debates about social justice and education policy [14]. Capability is the opportunity or potential to achieve and is seen as more important than the actual achievement or outcome [26]. Education is itself a basic capability as it enables the expansion of freedom and widens aspirations. Agency, one's ability to pursue the goals one values, are critical for the life one wishes to lead. The social world is never static and people may either act against or conform to prevailing norms as they respond to structural changes and development initiatives [27]. It is not about requiring compliance to programme’s goals in social change interventions, but building resilience by valuing people’s capacity to act and choose more beneficial and developmental options [28]. Adolescence is a time of intense cognitive development, including the ability to process and internalise information [29]. Acting on informed choices changes with biological age, hence the importance of adopting a prospective study design that considers both dropout and retention.

### Social norms theory

Social norms theory is used to understand why social expectations exert a strong influence on how individuals behave. In this theory, social norms are defined as people’s beliefs about what others do, and what others expect them to do. Individuals comply with social norms for a variety of reasons: group belonging, identity, or outcome expectations. One of the most potent compliance mechanisms, as well as one of the most studied, is social influence. Individuals anticipate rewards or punishment in case of compliance or non-compliance with the norm, respectively [30]. Social norms are thus often maintained by positive and negative social sanctions, and individuals’ behaviours are influenced by their anticipation of what will occur if they do, or do not, comply [31]. Social norms theory provides a useful starting point for a consideration of gendered normative expectations of girls in this context, but for a more nuanced understanding of how they act on pathways to girls’ school dropout, it is necessary to review gender theory in more depth.

### Gender theory

Gender is a ‘multilevel structure, system, or institution of social practices that involve mutually reinforcing processes at the macro-structural/institutional level, the interactional level, and the individual level' [32]. Gender theory emphasises how people are socialised into upholding existing gendered identities and associated inequalities [31]. As gender is multidimensional, different strands of gender theory have developed: economic decision-making theories consider gendered specialisation in domestic and market responsibilities, empowerment theories consider subordination of women across all levels, and performance theories look at the symbolic aspects and symbolic displays of gender [33]. Performance theory builds on insights from symbolic interactionist literature, in particular, a seminal paper on ‘doing gender' by West and Zimmerman [32]. This theory is based on the understanding that gender, unlike sex, is ‘learnt’ rather than biologically determined. Through everyday practices individuals ‘do’ or ‘perform’ gender, creating socially constructed differences between men and women which are systematically reproduced in social interactions and hence become seen as innate and of essential nature [29].

We have drawn on gender performance theory to better understand the data, following the findings of the 2005 India Human Development Survey analysis of what shapes decisions around marriage [33]. Observance of purdah (keeping women from the view of men they are not related to) and male-female separation in the household, as markers of gender performance, significantly influenced the decisions. Economic factors (availability of wage employment, dowry expectations, and wedding expenses) and empowerment dimensions of gender (women’s role in household decision making and access to and control over resources) did not. Where gender performance received less emphasis marriage got delayed, explaining a large part of the urban-rural difference in age at marriage [33].

In this paper, we adopt a prospective qualitative case study design to investigate gender socialisation in the context of SC/ST communities in Northern Karnataka. We analyse how gender-related norms interact with poverty and family background to result in girls’ dropout from secondary school. In addition, we identify the main facilitators of school retention and modifications to gender performance.

## Methods

### Study design and sampling

The data are from a prospective qualitative study, nested within a cluster randomized trial to evaluate the impact of *Samata*, an intervention intended to increase secondary school enrolment, retention and completion rates among girls from scheduled castes and tribes (SC/ST) in Bagalkote and Vijayapura districts in northern Karnataka, India [16,17]. Formative research among adolescent girls, their parents and teachers [34] informed the design of this multi-level intervention. It works with key stakeholders -low caste adolescent girls and their families, adolescent boys, village communities, school development and management committees (SDMC), policy makers and policy implementers—to change social norms and practices regarding gender, child marriage and girls’ education. It also links families to government schemes that provide scholarships, bicycles and other incentives to support girl’s retention in school.

This paper uses the first and second rounds of in-depth interviews with a subset of girls. The sampling of study participants was purposive based on age, academic performance, socioeconomic status, *Devadasi* background of their family, and distributed equally across both districts. Thirty girls were recruited from the list of girls selected for the trial, and the first round of interviews was conducted from March to June 2014 at a time when respondents were in 8^th^ standard and approximately 13 to 14 years old. Sixteen months later the same girls were approached for a second interview (between August to October 2015), with 27 girls available (the parents of one girl did not consent to her participation, and two others had migrated). It was decided to recruit an additional six girls whose mothers were dedicated as *Devadasi* to boost our ability to document both vulnerability and resilience among those considered particularly marginalised within the lower caste. Hence, 33 girls were interviewed in the second round. For this paper, we used information collected from 36 girls (30 girls from round one and six additional girls in round two).

### Data collection and analysis

**Fig 1:**
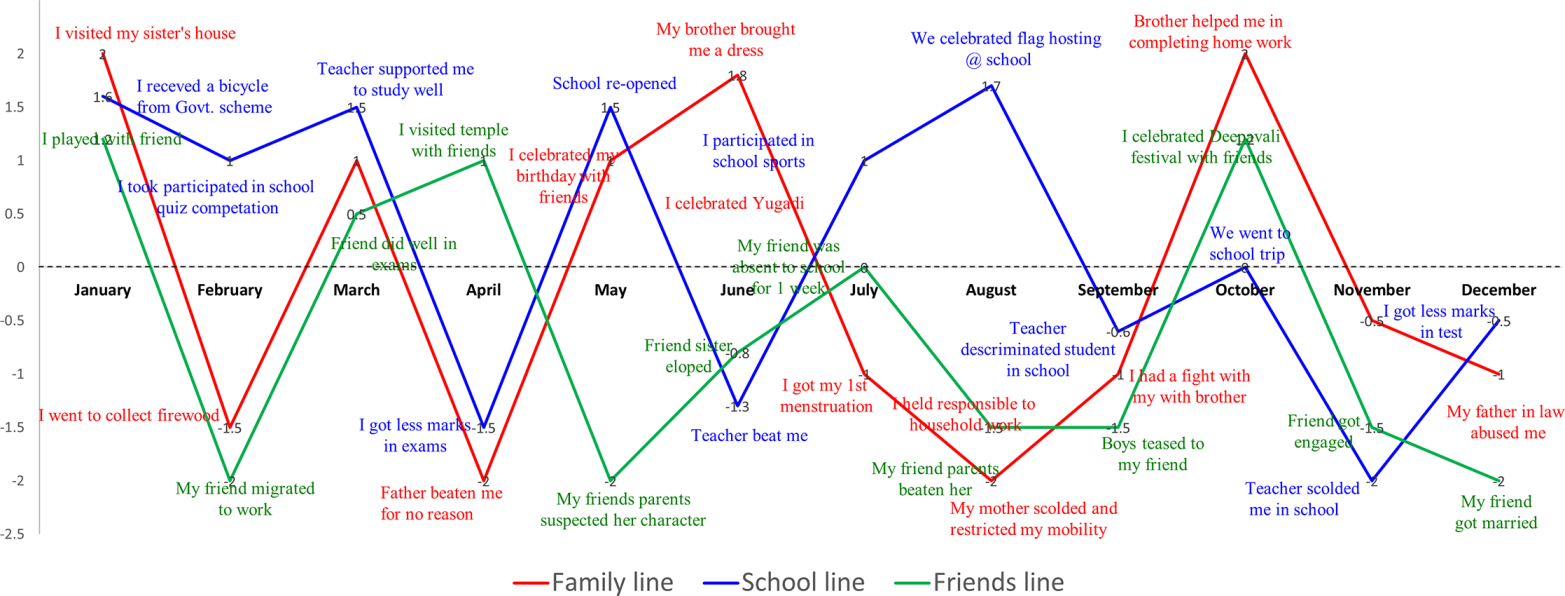
A sample lifeline chart as part of the study.

Data collection and analysis were iterative with the in-depth analysis of baseline interviews shaping the second round topic guides and tailored to the individual. Participatory tools facilitated rapport building, which was needed to obtain potentially sensitive information in a relaxed way. A life-line tool was used to mark events on a timeline to aid recall and focus the conversation on important and potentially sensitive issues [35]. Time was marked in months on the x-axis; events associated with positive feelings were marked above the x-axis, and those that brought up sadness or other negative sentiments were marked below the axis (see Fig 1). Using different colours, three main topic areas were plotted: significant events within the family, notable issues related to school, and specific events experienced by her friends. Analysing the first-round interviews identified gaps in our understanding of the family background, including the whereabouts, educational and marital status of the respondent's siblings. The information on family background was captured by letting the girls draw a family tree or genealogical chart, another helpful participatory tool [36] to improve rapport at the start of the semi-structured interview. The topic guide then explored school attendance patterns; support and encouragement for education received at home and at school; aspirations (local role models); family discussion of engagement and marriage, menarche, mobility restrictions and the occurrence of teasing. Apart from reporting personal experience, we enquired about school dropout and marriage among their peers and sisters. The lifeline tool was used throughout the interview to stimulate detailed discussion on the main topics.

The institutional review board at St. Johns Medical College, Bangalore, India; London School of Hygiene and Tropical Medicine, London, UK; and, University of Manitoba, Winnipeg, Canada has reviewed and approved this study. Written informed consent and assent was obtained from parents and girls respectively in their preferred language—either Kannada (the local language of Karnataka) or Lambani (the language spoken by the Lambani, a scheduled caste that resides in hamlets geographically separate from other villages). The main interview was preceded by two introductory meetings: the first one to explain the study and obtain written consent from the parent or guardian; and the second to explain the lifeline tool in a playful way using mock examples. After obtaining informed assent from the girl, the main interview was conducted in a place of her choice, which was usually her home. Every effort was made to interview girls in private with any interruptions by family members entering the room noted so that it could be accounted for during the analysis.

Two female investigators interviewed the girls in the first round, and they accompanied two new, more experienced interviewers, in the second round. All four investigators underwent intensive training on research ethics, qualitative research methodologies and interviewing techniques. To pilot the study tools, interviews were conducted with different girls to refine the wording and structure of the interview guide. All interviews were audio-recorded, transcribed verbatim and then translated into English. The first author reviewed all translated transcripts against the digital audio files for accuracy and completeness. To protect privacy, fictitious names are used throughout this paper.

We combined thematic and narrative analyses [37]. The thematic coding into primary and detailed subthemes was done using NVivo10 [38]. Thematic saturation was observed, and extensive memos written while coding which provided space to compare the data and to draw inferences and interpretations, based on those comparisons [39]. Initial thematic analysis allowed us to draw on individual cases primarily to represent themes across the sample. Case stories were constructed across both rounds of interviews, and our narrative analysis considers individual cases to understand their particularities. In writing up, we present data on those girls who have dropped out as short case studies to document cumulative influences of social expectations and family background. We then contrast the central themes and behavioural patterns which characterise the categories of regular school attendees and of those girls who seem on the trajectory of dropping out.

## Results

### Profile of girls participating in the case studies

With school dropout and retention as a central theme, Table 1 summarizes the socio-demographic profile of all 36 girls interviewed, according to their school attendance in round 2: six girls had dropped out of school, twenty were attending school regularly, and ten reported missing school frequently.

**Table 1:** Socio-demographic characteristics of adolescent girls interviewed by school attendance status (n = 36).

Characteristics	Dropped out n = 6	Regular attending n = 20	Frequently absent n = 10
**Age *(round 2)***			
14	1	0	1
15	2	11	3
16	3	9	6
**Caste or Tribe**			
Scheduled Caste	5	18	10
Scheduled Tribe	1	2	0
**Marital status *(round 2)***			
Engaged	0	0	1
Married	3	0	0
Not married	3	20	9
**First-born girl child**	4	6	6
**Father literate**	4	6	7
**Mother literate**	4	5	1
**Mother is *Devadasi***	1	6*	2

* recruited in the second round of interviews.

The three girls who were married by the second round of interviews had all left school. The one girl who was engaged reported missing school frequently.

We now turn to contextualising reasons for school dropout, drawing from the data accounts of processes and events leading up to leaving school.

### How and why girls dropped out of school

Six girls left school before the second interview. One girl got married and moved to her in-law's, and another one migrated to a neighbouring state for work. The other five were available to narrate the sequence of events and perceived reasons for drop out.

#### Case # 1 –Sheela

Sheela from a village in Bagalkote was 14 and enrolled in 8th standard during the first interview in April 2014. Her father worked in a private firm, and her mother made and sold broomsticks. Sheela had two younger brothers and one elder sister, who discontinued school soon after getting married to her maternal uncle at age eleven to reduce the family burden. Sheela had missed school regularly due to her workload at home: domestic chores, caring for her brothers and supporting her mother’s business. She had described her father as supportive of her education though not her mother, who prioritised schooling of the brothers, who in contrast to herself, were in private school. While she had not performed well, she viewed school and the teachers positively and had received all government entitlements (midday meal, books, bags, uniform and scholarship money). In the second round, Sheela recalled how being mistaken for her best friend had led to her leaving school:

*“One day, we both were on the way to school, and the same boy came on a bike and started discussing with my friend … later she went with him … my uncle observed my friend and me talking to the boy, and he mixed us up. He told my father that I went on his bike … my father initially did not believe my uncle, but he started suspecting me … he discussed with my mother who also did not believe it … but the suspicions were on their mind. One day my father suddenly said from now on you don't need to go school … he said that we both are going out for work, and someone has to be there to look after the house. But that was not the reason, he didn't disclose his real fear … his fear was me … if I fall in love or elope with a boy then it will affect my marriage and also the family honour … so that he hasn't agreed to send me to school … I was a scapegoat and left the school …I was helpless to convince my father" (Sheela, Bagalkote, Father: pre-university college & Mother: 4^th^ class)*.

This quote shows that in interacting with boys, Sheela is considered by people in her community to be acting incorrectly for a girl of her age, warranting their report of her perceived ‘misbehaviour’ to her father. The fear of Sheela attracting further gossip, and other social sanctions, is enough to drive her father to force her to drop out of school. It is feasible to assume this reflects a broader societal fear of girls transcending normative gendered expectations, and that going to school may enable this. For Sheela, the ‘unspoken’ fear about her ‘incorrect’ gender performance topped other gender biases within the family, which could have led to her school dropout: the imposition of strict mobility restrictions after menarche, lower aspirations for girl’s education, and disproportionate burden for girls to support the household’s livelihood, leaving less time for study and attending school.

#### Case #2 –Mahadevi

Mahadevi was the eldest of 4 sisters and a brother with illiterate parents who struggled to make a living on the small piece of land they owned. At the time of the first interview, age 15, she was in 8^th^ class and a good student. However, her responsibilities as eldest daughter compromised her school attendance:

“*Sir used to say*, *don’t be absent but these people [parents] said don’t go to school*. *My father used to threaten me; he used to say that there is work in the house and how it will get done if you keep going to school* … *" (Mahadevi*, *Bagalkote*, *both parents are illiterate)*.

Mahadevi started caring for her siblings when she was six and took up agricultural labour at the age of ten, mainly during school holidays. She did household chores and looked after the animals. She perceived her parents as discriminating against her.

“*My parents believe that girls will go to their husband’s house whereas boys will look after their parents in their old age* …*so they give preference to a son as compared to their daughters” (Mahadevi*, *Bagalkote*, *both parents are illiterate)*.

Her father’s alcohol abuse resulted in constant family conflicts. However, she was able to complete the 8^th^ class with good marks in all subjects and joined the 9^th^ class, yet her fate changed as she recalled in the second interview. Mahadevi described how a boy from another village had taken a liking to a girl in her school, leading to an unsanctioned marriage. The story and associated panic about other girls doing the same spread quickly in the community, and a few months later, Mahadevi’s parents argued that something similar might happen to her. This would adversely affect their family since they had three more daughters [who need to get married], leaving her with a sense of helplessness and frustration:

*"I didn't want to drop out from school, and he said no you have to get married, so you stop going to school … I found it difficult to think that I am leaving school …I was helpless. I said I don't want to get married, but still, they got me married" (Mahadevi, Bagalkote, both parents are illiterate)*.

Her parents were presented with an opportunity when her maternal uncle separated from his first wife and showed his willingness to marry Mahadevi–the wedding was arranged immediately. Initially, while still living at home, she continued school. However, this soon met with the opposition of the husband and in-laws who demanded that she come and live with them. The gender-related practice of girls moving in with in-laws, transferred the decision regarding her well-being and development from parents to husband and in-laws, maintaining Mahadevi’s lack of control over her own life.

#### Case #3 –Pooja

Pooja was 15 years old at baseline and had joined a new school for 8^th^ class. As the eldest of three sisters and two brothers, she lived with her *Devadasi* mother and her father was employed in a nearby hotel. She was very positive about her teachers and her school, equipped with a playground, library and a separate girls’ toilet. She obtained government assistance including books, a bag, a uniform, a bicycle and a scholarship. During the second round of interviews, she narrated:

*“I completed 8^th^ standard and joined the 9^th^ class. My mother was suffering from a heart problem, and no one was there at home to care… so I used to miss school regularly. But my mother died when I was in 9^th^ class and how can I think of going school in such situation. I was solely responsible to care for my sisters and brothers. I left school and started caring for them… looking after the house and working outside” (Pooja, Bagalkote, Father: 6^th^ Class & Mother: 5^th^ class)*.

Upon her mother’s death, Pooja inherited the role of full-time caregiver, which she perceived to be both ‘natural’ and beyond questioning. There was no mention of any reputational concerns in her narrative–her drop-out was due to family circumstances and the instrumental need for her labour.

#### Case #4 –Gayatri

At the first interview, Gayatri was 14 years old and in 8^th^ standard, the last class available in her remote village. Her elder brother and younger sister were both studying in a residential school in a nearby town. Both parents were illiterate; her father was a fisherman and her mother a labourer. Despite the family's limited resources, the children were never expected to contribute to the household income. Gayatri’s domestic duties did not affect her studies: she was a high-performing child and rarely missed school. During the second interview, she recounts the changes that came with the onset of menstruation including the traditional menarche ritual where she was confined to the house for eight days, and the various subsequent restrictions imposed on Gayatri’s dress, food habits, and mobility:

*"… earlier I could go anywhere*, *but now I can't do that* …*now they say*, *you become doddaki (grown up)*, *don't wander here and there* …*they restrict me to play with boys* …*if I speak to some they say don't stand and speak like that*, *you are not small* …*they scold if I wear short dress* … *a lot of restrictions and I feel bad about it" (Gayatri*, *Vijayapura*, *both parents are illiterate)*.

Since menarche signals the start of womanhood, avoiding any accusation of unchaste behaviour became a preoccupation. One day, Gayatri’s parents arranged an informal engagement ceremony with her maternal uncle, and soon after they performed a *yadi-mele-shadi* (marriage on the spot after an instant decision) at the age of 14, Gayatri was largely unaware of the significance of the event. Although she had been married for 18 months, she still lived at home as *gauna* had not yet been performed (the ritual at the time the girl moves in with the husband, and the marriage gets consummated). While both her parents and in-laws initially agreed that Gayatri should continue her education, she dropped out after completed 8^th^ standard. She failed to convince her parents to let her attend 9^th^ standard, available only in a nearby village:

*“…I think the society won't keep quiet if my parents send me to school after marriage … they blame my parents … that would be the reason for my parents not to send me to school… I wanted to go to school … that was my dream … but they did not value my wish” (Gayatri, Vijayapura, both the parents are illiterate)*.

The fear of social sanctions and avoiding any suspicion of her sexual purity was the main reason for Gayatri’s drop out. Her parents have prioritised ensuring Gayatri adherences to mobility restrictions over her schooling.

#### Case #5 –Sharanamma

As the fifth of six siblings, Sharanamma leaving school seemed predictable given the pronounced gender bias in parental aspirations and discriminatory practices in her family. Her father–educated to 3^rd^ standard and working as a shepherd–did not approve of her mother working as a cook in a student hostel and this had led to many fights between her parents. The first two daughters left school early and married at a very young age to two brothers to reduce wedding expenses. While her elder brother received on-going family support for his education, the fourth child, a daughter, got married when she was in 8^th^ class. Sharanamma’s parents had felt compelled to accept a marriage proposal by the bridegroom’s family. Although they had promised the sister she could continue school, her in-laws did not consent, so she dropped out. The parents never showed interest in Sharanamma’s education, and she shouldered a heavy burden at home:

“*After getting up at 5 am daily*, *I clean the vessels*, *broom [sweep] the floors and after that*, *I go to the canal to wash the clothes*. *I cook food and look after my elder sister children*. *My father asks me to go to the field to graze sheep whenever he goes out* …*mother asks me to cook food* …*it is a lot of work*, *and I can't concentrate on my studies” (Sharanamma*, *Vijayapura*, *Father*: *6*^*th*^ *Class & Mother*: *illiterate)*.

Her regular absences from school and inability to do her homework resulted in difficulties keeping up and punishment by her teachers.

“*Our teachers scolded me* … *they [teachers] said that if I can’t complete homework*, *I should sit at home instead of coming to school* … *I told them I have a lot of work at home*. *Even then*, *they punished*. *They use to beat me with a stick* … *my hands became red” (Sharanamma*, *Vijayapura*, *Father*: *6*^*th*^ *Class & Mother*: *illiterate)*.

Lacking support at home and at school, Sharanamma lost interest in school and left after 8^th^ standard.

In summary, these five case studies demonstrate a combination of factors leading to girls dropping out of school. While Mahadevi and Gayatri initially continued education following early marriage, this was short-lived, even before joining their husband’s family. The expectation of girls to help out at home seemed primary in Pooja's and Sharanamma's case, while for the other three, concerns about reputational risks were the final decisive factor. All girls came from large families (except Gayatri), and parental aspirations for girl's education were low, though this was not true initially for Pooja and Gayatri. For all six girls, and as evident from narratives in the first round of interviews, the trajectory to school dropout began with their frequent school absence, or their parents’ lack of aspirations for their daughter’s education.

We first contrast the backgrounds of regular school goers from those who left, before turning to those potentially on a trajectory to drop-out as they reported frequently missing school during the second interview round.

### Regular school goers

Compared to the girls who dropped out, the 20 girls who attended school regularly had parents who placed a high value on education, and had more harmonious family backgrounds, allowing more flexibility around gender expectations. More open interaction at home translated into more assertive communication at school.

### Parental aspirations

There was little evidence of gender discrimination in the narratives of girls who rarely miss school, with parents encouraging education for all children and extending their support for schooling regardless of economic and educational background.

“*Sometimes there was no money …I get frustrated… my father works hard to give us education… despite their difficulties; they never make us feel bad… they never share their financial problems with us…I feel proud of them” (Usha*, *Bagalkote*, *Both the parents are illiterate)*.

Most parents were engaged in the girl’s educational accomplishments, monitoring her academic progress and interacting with teachers. Five out of 20 girls interviewed talked about a parent helping them complete school assignments, and others drew support from elder sisters or brothers to perform well in their studies.

*“My mother doesn't know much, but my father helps me in completing my assignments and homework. My sisters help if there is drawing to be done … everyone in the family supports me very well. Once I had scored less in unit tests and father scolded me and said I could have done better… my mother always advises me to study well” (Radha, Bagalkote, Father: graduate & Mother: illiterate)*.

Parents encouraged their daughters to score well in exams and had career aspirations for them. In response to questions regarding role models, girls knew someone of higher social status with a ‘good’ job among their relatives or acquaintances, who they described as inspiring them. All mentioned having their own career goals, which commonly aligned with wanting to fulfil their parents’ wishes by becoming a doctor, district commissioner, lawyer, teacher, bank manager, etc. Girls with mothers from Devadasi backgrounds communicated that their parents wanted their daughter to have the opportunities that they had been denied:

*“She [mother] says that my life should be unlike hers. She wants me to be highly educated so that I can have a worry free life ahead… after listening to her words, I feel happy and highly motivated, involving myself in studies” (Preeti, Vijayapura, Father: 3^rd^ class & Mother: illiterate)*.

These girls also had ambitions and interests that went beyond education and career, and girls in this group enjoyed their parents’ encouragement in developing other skills in dance or sport. They could perform at school functions or take part in inter-school sports competitions.

### Communication and cooperation in familial relationships

In their narratives, girls described supportive and harmonious family environments with no mention of alcohol problems. Extensive communication characterised these families, and girls described their parents as open to hearing their daughter’s concerns and guiding them on safety issues including how to cross the road safely, how to interact with strangers, and how to deal with teasing from men and boys. One girl said, *“they say*, *go school without tensions and if any boy tells you something then come and tell us…we will take care of it" (Girija*, *Bagalkote*, *Father*: *pre-university college & Mother*: *illiterate)*. Girls talked about the importance of trust in the parent-child relationship. Strong educational aspirations combined with open communication within the family to develop greater levels trust.

“*If the parents have faith in their children then they will send them anywhere*, *if they don’t trust them then how can others*? *Because of this*, *they [parents] need to question themselves*. *Sometimes their opinion might be wrong” (Sudha*, *Bagalkote*, *Father*:*10*^*th*^ *class & Mother*: *6*^*th*^ *class)*.

Parents did expect girls to perform household chores, often from a very young age. This was often considered as helping girls prepare for their futures. One girl said, *"my mother says if you don't know the domestic work…then your in-laws will blame us later (Rutu*, *Bagalkote*, *Father*: *pre-university college & Mother*: *illiterate)*. Many had looked after siblings, but this did not interfere with their studies. There was no mention of parents migrating for work, leaving children at home. Girls commonly described assisting their mother with household work, rather than absorbing her household responsibilities alone.

Better coordination and cooperation to share the burden of household tasks is often described as taking place in households where education for girls is prioritised. For example, some girls described assisting their parents with agricultural work during holidays, and taking care of livestock after school hours, and in return their mothers taking over a greater share of responsibility for such tasks during exams.

*"As soon as I come from school, I do the household chores, and then I sit at around seven till ten o' clock in the night and write and complete my assignments … I don't do any [household] work during exam time, and my parents are okay with it” (Asha, Vijayapura, Father:10^th^ class & Mother: illiterate)*.

Girls in this group perceived teasing as a major factor contributing to drop out of girls in their community and pointed to the importance of family support, in helping them manage harassment.

*“a boy used to come to my school… with his friends, he was coming and standing nearby our house… he tried to touch me once while I was going school… after that, I informed the situation to my mother, and she scolded and warned him… so then he stopped doing that" (Kalavati, Bagalkote, Father:10^th^ class & Mother: illiterate)*.

### Support and communication at school

Most girls felt supported at school with only two girls talking about the unfair treatment by some teachers. Some girls reported that teachers punished girls who didn't complete assignments on time or who missed school frequently. Good communication and coordination practices at home translated into how girls conducted themselves at school, enabling them to confidently interact with teachers. They were also actively involved in extra-curricular activities in school. For most, attending school had a valued social function.

*"It gives great pleasure of having the company of friends in school. I feel bad when not attending school …I skip the classes only if it is inevitable like having ceremonies or function at home…otherwise, I never skip” (Rekha, Bagalkote, Father: graduate & Mother: illiterate)*.

In this group, absence from school was infrequent, of short duration. When absence did occur it was due to illness, funerals, family or religious functions. These girls communicated anticipated absences, informing teachers in advance of them taking leave and afterwards consulting friends to help them understand the material they missed during their absence. Two girls did report some longer-term absence from school in the past, attributing it to lack of interest and some belief in superstition. In both cases their families had intervened, to ensure their daughter return to school and continue their education.

As will be elaborated in the next section, even among girls who regularly attend school, menarche rituals are observed as significant events, which cause them to miss large periods of school. In this group, all 20 girls underwent menarche rituals, missing school for its duration: five days for most, while for four girls the celebration and formalities took around two weeks, with regular school attendance resuming afterwards. Only one girl mentioned her family discussing matrimony at this time, with a marriage proposal being postponed. All families imposed mobility restrictions on the girls when she attained puberty.

### On the brink of dropout?

After contrasting the backgrounds of regular school goers from those who had dropped out, we now consider girls who seem to have started the trajectory of dropout. The narratives of the ten girls who reported missing school frequently are strikingly different from those who attended regularly, and are outlined below.

### Economic and household responsibilities

Domestic and caring duties and agricultural labour were the prime reasons for the girls missing school, though spiritual ceremonies, family functions, illness and monthly menstrual periods also led them to miss classes. Gender asymmetries in these families were stark, with parents not valuing girl's education, in part because they perceived it as lacking potential for economic returns. Rather, families followed a division of labour based on socially recognised gender roles burdening girls with household chores, fetching water and bringing firewood from the forest for more than four hours a day. These girls had started looking after their siblings at the age of 6 to 8, a particular burden when parents migrated for work leaving the children behind. Some were responsible for looking after livestock and work as an agricultural labourer.

While families received most government entitlements, some girls reported their parents utilising their scholarship money for their own purpose. The economic and social realities at home deprived girls of agency both at home and at school.

Absenteeism patterns varied among respondents: while frequent for everyone, not all had prolonged periods of missing school. Two girls were forced to miss school every Saturday, because the earlier school start time on this day did not allow them sufficient time to finish their household responsibilities. Four girls were absent for almost two months a year during the sowing and harvesting season:

*"I used to go to school only on every alternative day in January, as I went to cut sugarcane in my uncle's farm. I couldn't attend the school for fifteen days or more … again I missed classes in August as I went to harvest Jola [sorghum]” (Geeta, Bagalkote, Father: pre-university college & Mother: 5^th^ class)*.

### Lack of parental support

Girls in this group reported very little encouragement from parents, who showed little or no interest in their achievements at school. Eight girls reported attempts by their fathers to make her leave school, with some support from their mothers allowing them to continue albeit intermittently. Many reported alcohols abuse by the father.

*“They don’t support me; in fact, they want me to stop going to school. No one is there to encourage me that I should attend school…My father takes even my petty savings for his habits…my mother tells me that ‘haasige iddaste, kaalu chachabku’ (stretch your legs only as much as the bed allows you to) and they blame me and my education for the fact family is struggling this much… I feel sad, and I do not say anything … I just stay mute” (Bharati, Vijayapura, both the parents are illiterate)*.

With less harmonious family backgrounds, there was no scope for girls to share feelings or concerns–there was no communication. Missing school frequently, and having taken on a mature role at home from a young age, also meant less involvement in extra curriculum activities, sports, recreation and contact with friends.

### Coming of age and marriageability

Turning from poverty-related reasons to those related to potential reputational damage, a clear divergence started at menarche, with its prolonged rituals. One of the girls did not attend ninth standard for almost two months because of menarche. The rituals families perform to celebrate her menarche vary in length, from five to as many as twenty-one days.

*“As soon as it [first menstruation] happened, they made me sit for nine days. They applied turmeric paste to my body and gave bath using neem leaf…. They dressed me up with sari and performed arathi [Hindu religious ritual of worship, a part of puja] … older women sang … they gave me dry coconut with jaggery and sajaka [a sweet dish made of wheat and jiggery] to eat every day. All my relatives, cousins and neighbours came to bless me and gave a gift…it went like this for nine days … They don’t allow me to go out for all nine days and I stayed away from school even longer” (Shruti, Bagalkote, Father:10^th^ class & Mother: illiterate)*.

The local Kannada terms for menarche are *dodavaladlu*, *maineradalu* and *rutumatiadlu* with explicit connotations of the girl maturing, attaining womanhood and her ability to give birth. Celebrating menarche seemed like a public announcement of girl’s readiness for marriage.

Unlike the parents who prioritised education for their daughters, in these families, parents started talking about marriage soon after menarche. A good marriage for their daughters seemed the highest aspiration of these parents, after which they are released from taking financial responsibility for their daughters. Parents aim to secure marriages for their daughters by ensuring the maintenance of their reputation. For many girls, the possibility of suspicion and doubts about her moral conduct or reputation were linked to her school attendance. Preventing girls from going to school, therefore, was portrayed as a method used by parents to uphold their daughter’s reputations.

*“Our parents think their girls are like Kannadi [mirror] …if it breaks once it can't be put back together … means, if they go school and don't study … if they fall in love with someone else. . . . run away …it brings a bad name to the parents …to their family. So they have big anxiety to support girls schooling” (Kavya, Vijayapura, Both the parents are illiterate)*.

Apart from perceiving school as an area of potential danger to their daughters, parents were keen to avoid societal judgement of the girl as characterless for being seen in ‘male' public spaces. Her safety and sexual purity is a priority for many parents, causing them to restrict her mobility and visibility in public spaces a priority.

*“They say, don't go to the bus stand. Don't go shopping, don't wander around the village. Mostly they say don’t go near the bus stand …because men are not alright, with that fear only they raise girls. So mother and grandmother say men are not good so why you wander in the village” (Seema, Bagalkote, Both the parents are illiterate)*.

Some girls consider these restrictions positively, accepting and endorsing this practice, understanding their parents had their well-being in mind. Others expressed feelings of deprivation about their limited social space, and felt it affected their confidence and knowledge. It was an obstacle to their continued education as the commute to ninth standard in a different village was seen as too much of a risk to their reputation. Not only school but recreation was affected as well:

*“After my puberty grandmother, father, and mother say ‘you are older now … don't move here and there …stop playing games'. So I left it …my parents told me not to play with children because they thought that the neighbours could say something … I left playing games” (Sumathi, Bagalkote, Father: illiterate & Mother: 7^th^ class)*.

These girls frequently mentioned the opinion of neighbours and close relatives, who always seemed ready to blame the girls for either neglecting gender-specific roles at home by going to school, or by raising suspicions around her character when she stayed at home.

*“They discuss themselves that what could be the reasons for me to miss the classes on that day. They think there might be something wrong, like this, they think. They might suspect illegal relationships or fall in love with boys. They doubt. They don't think that it would hurt us a lot if they believe so” (Ramya, Vijayapura, Both the parents are illiterate)*.

Girls in this group denied experiencing teasing by boys or men, but all mentioned friends who had been affected. They explained it was not the act of teasing itself that affected girls’ schooling but parents’ anxiety about her falling in love and running away with a boy. Unlike the families where education was prioritised, there was no scope for these girls to discuss harassment with her parents, for fear they would turn the blame on her. In such home environments teasing became unspeakable, and hence the only option was denying it happened to her. There were no conversations about educational and career aspirations, and school teachers were mentioned as the primary role model.

### Negative experiences at school

All ten respondents expressed sadness and disappointment about not attending school regularly and wanted to go back. While most did not fault the teachers, they expressed fear of punishment.

*"I feel scared to go school after missing sometimes… our sirs don't distinguish between boys and girls and they just beat… ask me to stand in the sun…. they used to shame me in front of other students… I lose interest to go to school and felt like leaving school” (Sharada, Bagalkote, Both the parents are illiterate)*.

They felt dejected by teachers scolding them for staying away from school and not performing well, as they had difficulties understanding lessons. They missed the social connections and were losing friends by not being in touch for days on end.

These girls were more likely to mention their monthly periods as a reason for missing school. Many girls described ‘choosing’ to leave school due to the lack of sanitary facilities, and having only male teachers in the school.

*"I don't go to school for those five days. . . . I experience abdominal pain … we don't have toilets in school … we feel uncomfortable to ask our sirs, and so I feel better to stay back at home for those days" (Vijaya, Bagalkote, Both the parents are illiterate)*.

The families of these girls were slightly larger than those of girls who attended school regularly (about four siblings)–see Table 1. Elder sisters had set the patterns of drop out for the girls ‘on the brink’: six out of seven had dropped out before 10^th^ class, whereas among regular school goers only 3 out of 22 elder sisters did.

## Discussion

This prospective qualitative study has analysed how gender norms interact with poverty and family dynamics on girls’ pathways of dropping out of secondary school. Among SC/ST communities in Northern Karnataka, as young adolescent girls go through a period of intense gender socialisation, three inter-related gender norms constrain girls’ agency, and likelihood of school dropout. They relate to protecting girls’ sexual “purity” by limiting freedom beyond the home; their domestic role in household and caring duties; and the lower priority given to education of girls compared to boys. Our data has shown how poverty exacerbates such discriminatory gender practices, and increases the likelihood of parental conflict and alcohol abuse in girls’ homes. However, we have found that rather than poverty alone, the two most important factors influencing whether a girl attends school regularly communication between parents and daughters, and the value placed on education by parents. Our data confirmed that dropout was not a one-off event, but rather a process resulting from strong social norms at community level interacting with household level deprivation, influencing relationships at home, school and on the way to school [13].

Girls who attended school regularly had parents who actively encouraged their educational and career aspirations. While such girls did engage in domestic and caring roles, this was not at the cost of their education. Family dynamics were characterised by open communication between parents and children and cooperation allowing girls to better balance schoolwork with domestic chores. This fostered the girl’s agency to communicate not only at home, but importantly at school too. This high level of communication and the shared aspiration in pursuing education left more room for girls to negotiate subtle changes to gender performance in public spaces. Trust seemed to replace suspicion and girls could count on parents’ support when she experienced harassment. Both parents and daughters could resist pressures of social sanctions and pay less attention to “what others in the community do” or “what others expect them (or their daughters) to do”.

Education is beginning to replace marriageability of daughters as a route to family honour and respectability, at least in parts of the community. In recent times of fast economic change, tensions have been observed throughout India between status attainment through the performance of ‘modernity’ and status attainment through an opposing, acceptable gender performance of girls, characterised by a focus on morality and “purity” [33]. While the ideal of girls’ virginity before marriage remains unchanged, some of its manifestations have relaxed over time. We have seen how the strength of a potentially harmful norm was thus weakened by a new aspirational norm of education, showing that social practices are malleable. Gender norms, or compliance with gender norms can be modified, despite the pressure of social expectations. We see this in our interesting finding that parental education was less important than parent’s aspirations for their daughters’ education, in influencing decisions about girls’ school dropout. This reflects a change over time in beliefs about what girls can and cannot do. Our findings seem in line with a prospective cohort study in Andhra Pradesh, where a clear link was established between educational aspiration of parents, their children’s aspirations and educational achievement, with individual agency regulating aspirations [40].

The data also showed how girls in deprived households were forced to take increased responsibility for domestic duties, and parents prioritised son’s education, as is common throughout India [41]. As there was little communication within the families, girls’ narratives alone cannot tell us whether the family’s need for their labour was seen as gender discrimination *per se*. This could alternatively reflect a sensible investment on the part of parents, and recognition of structural gender inequalities in labour markets and the perception that sons will look after their parents in old age. While the study population is characterised by social and economic disadvantage, it is notable that girls on, or at the end of, the trajectory of dropping out were more commonly from particularly deprived families. This reflects findings from the *Samata* baseline survey, where there was a significantly higher likelihood of school dropout and frequent absenteeism among girls from economically disadvantaged households. Moreover, girl’s involvement in paid work, commonly in response to family poverty, was strongly associated with frequent absenteeism [42]. In our study, poverty has been shown to strongly affect agency, especially when considered as deprivation beyond a lack of resources. Families who faced difficult decisions regarding their livelihood also appeared to have constrained choice in decisions about whether to remove their daughters from schooling. This is arguably due to fears of contravening normative community expectations regarding the behaviour of young girls, and damaging their reputation in doing so. The socially disadvantaged individual is more susceptible to follow harmful social practices due to lack of voice and social security as well as denial of choices and opportunities [14,43,44].

Using a capabilities lens has helped us consider how girls perceive and experience barriers to schooling. Long travelling distances and inappropriate sanitation facilities at school were mentioned by girls from deprived backgrounds. In contrast, girls from supportive families were able to overcome these structural barriers at, or on the way, to school. When agency was nurtured at home, girls were able to get more out of schooling, further developing their capabilities. This has interesting implications given the findings of a qualitative study analysing school retention in an African setting found that if not at home, girls’ aspirations could be generated and nurtured by other key people, a teacher at school or someone in the community who helped them realise their capabilities [14].

Returning to the findings of Desai and Andrist (2010) using the 2005 India Human Development Survey, our analyses underlines the strong influence of expectations for girls to display a ‘correct’ gender performance in shaping decisions about both marriage and school attendance, especially at the intersection with poverty. Where parental educational aspirations for daughters were weak or absent, parents invested more intensely in enforcing correct performance of gender, aiming to secure her future, and perceived well-being, in a good marriage. The lack of communication resulted in family cultures of silence precluding girls from sharing any harassment they experienced for fear of bringing suspicion onto themselves. It is notable how poverty-related arguments dominated the narratives of girls ‘on the brink’ of dropping out of school, while reputational gender performance remained more ‘unspoken’. This may have been due to the fact that girls endorse mobility restrictions as they internalise these practices as being ‘for their own good’. Gender inequalities are ‘naturalised’ [31] yet fears of girls’ parents or other people in the community suspecting girls’ characters loomed large. In contrast, several of the girls who had dropped out and experienced the full impact of social sanctions in doing so, did identify the “purity” norms as a ‘real’ reason for dropout. Reputational fears were, in these cases, the deciding factor for dropout and was directly associated with early marriage. Like school dropout, marriage itself was a process with a delay in the second step of *gauna*. While education initially continued, parents’ concerns regarding safeguarding her sexual “purity” before marriage were reinforced after marriage until *gauna* was performed. While the delay of sexual relations and reproduction is positive, there seemed little scope for the girl to develop her agency as her education was curtailed.

### Limitations

This analysis does not include parental perspectives and therefore misses important information on what informs the decisions they make and barriers they perceive that influence girl’s school dropout. It is among the most deprived families that details on parent’s agency are especially lacking, due to girls’ self-acknowledged lack of communication with their parents. Additionally, the Samata intervention is targeting lower caste rural girls only, it does not allow for a full consideration of gender and poverty intersect with caste and residence. With only three married girls included in the study, we are also unable to comment fully on the interplay between marriage and school dropout. As girls were in ninth standard during this round of data collection, their full trajectories are not represented here, and it is likely that barriers to retention in the last year of secondary school will be different. This will be explored with the third round of interviews.

### Implications for interventions

Our research has implications for interventions, which aim to improve gender equality and educational outcomes in poor communities. Implications are presented at three levels: those at State and national level take into account structural issues; at family and community level; and school-based interventions.

Support at the national level requires the convergence of laws and policies related to health, and social and economic development. As poverty exacerbates gender inequalities and increases the risk of school dropout, outreach activities need to target economically deprived households proactively and provide support to ensure these families have access to all available government schemes and benefits. As dropping out from school is a process, the interventions should develop a strategy to identify the vulnerable households and plan for an intervention at multiple time periods to positively impact, and support families and girls to avoid the dropout process.

Promoting positive, aspirational norms regarding open communication between parents and children is central to developing stronger relationships of trust and confront family level drivers of school dropout. Family centric interventions are needed to help individuals reflect on issues of “purity”, honour and respectability, and how these could cause harm to girls and communities, and community campaigns should provide a platform to give visibility to parents and girls who modified gender performance without losing respectability. Girls agency needs to be built through peer group reflections where girls can meet and share their challenges and success. Girls from those families with educational aspirations can act as role models for others. To create a more conducive environment for girls’ education, peer group activities with boys to reflect on gender socialisation are important, and have shown promise elsewhere in India, for example through the medium of sport [45].

Girls who frequently missed school expressed their anxieties about punishment and discrimination by teachers. The role of the intervention can be two-fold: providing extra tuition classes to frequently absent girls, where they can regain some confidence; and providing training to the teachers on dealing with frequently absent and poorly performing students with more sensitivity. Interventions in schools where teachers can play an important role in developing agency of girls and promote gender equity at school could encourage girls’ aspirations and motivation to stay in school. Better tracking of attendance of girls in school can help interventions provide more immediate support to vulnerable girls and families.
